# Supplementary material for: The evaluation of a tissue-engineered cardiac patch seeded with hips derived cardiac progenitor cells in a rat left ventricular model
Source: PLoS One. 2020 Jun 8;15(6):e0234087. doi: 10.1371/journal.pone.0234087 (PMC7279601; doi:10.1371/journal.pone.0234087)
Supplement: Attachment — Submitted filename: Matsuzaki_patch 122219.docx [file pone.0234087.s001.docx]

Matsuzaki and colleagues submit their manuscript entitled, “The Evaluation of a Tissue-Engineered Cardiac Patch Seeded with hiPS Derived Cardiac Progenitor Cells in a Rat Left Ventricular Model” for consideration of publication in PLOS ONE. The authors use a rodent model to test whether a cell-seeded biodegradable tissue-engineered patch provides beneficial effects in LV function. The experiments are well designed with appropriate controls. Unfortunately, but not surprising cell-seeding of iPS cells did not lead to an increase in cardiomyocytes. The authors do demonstrate that a biodegradable patch can be used as a viable option for ventricular rupture.

Comments

1. The potential benefit of the biodegradable patch was the ability to cell-seed the patch. However, since the cell-seeding did not lead to increased myocardial cells, authors should clarify the benefit of their patch over non-absorbable patches.
2. Inclusion of a non-biodegradable patch as a comparison to the biodegradable patch could have been included. Do the authors have any comparison data? Especially in light that at 36 weeks, there was no difference between cell-seeded and no cell-seeding. So is a biodegradable patch add benefit?
3. Was vascularization assessed? Also, was staining performed for fibroblasts?
