# Supplementary material for: The evaluation of a tissue-engineered cardiac patch seeded with hips derived cardiac progenitor cells in a rat left ventricular model
Source: PLoS One. 2020 Jun 8;15(6):e0234087. doi: 10.1371/journal.pone.0234087 (PMC7279601; doi:10.1371/journal.pone.0234087)
Supplement: Attachment — Submitted filename: Review Comments to the Author newest.docx [file pone.0234087.s002.docx]

**Review Comments to the Author**

Please use the space provided to explain your answers to the questions above. You may also include additional comments for the author, including concerns about dual publication, research ethics, or publication ethics. (Please upload your review as an attachment if it exceeds 20,000 characters)

Reviewer #1: Matsuzaki and colleagues submit their manuscript entitled, “The Evaluation of a Tissue-Engineered Cardiac Patch Seeded with hiPS Derived Cardiac Progenitor Cells in a Rat Left Ventricular Model” for consideration of publication in PLOS ONE. The authors use a rodent model to test whether a cell-seeded biodegradable tissue-engineered patch provides beneficial effects in LV function. The experiments are well designed with appropriate controls. Unfortunately, but not surprising cell-seeding of iPS cells did not lead to an increase in cardiomyocytes. The authors do demonstrate that a biodegradable patch can be used as a viable option for ventricular rupture.
Comments
1. The potential benefit of the biodegradable patch was the ability to cell-seed the patch. However, since the cell-seeding did not lead to increased myocardial cells, authors should clarify the benefit of their patch over non-absorbable patches.

**Thank you for pointing out. The discussion section has been modified. (Page 14 line 227-Page 15 line 244)**.**Current non-biodegradable material which is used for LV repair has the risk of thrombosis and the need for frequent reoperation due to infection. Bioabsorbable materials are more useful than others as they serve as scaffolds for ingrowth of functional host myocardium and once degraded there is no longer a foreign material that can act as a nidus for infection.**

2. Inclusion of a non-biodegradable patch as a comparison to the biodegradable patch could have been included. Do the authors have any comparison data? Especially in light that at 36 weeks, there was no difference between cell-seeded and no cell-seeding. So is a biodegradable patch add benefit?

**Thanks for asking a question. As stated in question 1, we thought that bioabsorbable materials are useful for models of left ventricular formation after myocardial infarction. The design of this experiment focuses most on demonstrating the efficacy of iPS cell seeding using the bioabsorbable material. Therefore, the control group is only the non-seeded bioabsorbable material group. (page14 line 227-page15 line 244)**

3. Was vascularization assessed? Also, was staining performed for fibroblasts?

**Thank you for pointing out. We decided to evaluate angiogenesis in one-month and 6 month specimens by immunohistochemisty of CD31. It was newly confirmed by CD31 that the number of new blood vessels was higher in the seeded group. We did not stain for fibroblasts. The reason was difficulty in using immunohistochemical markers specific to markers and not other cell types capable of ECM production. (Page13 line 213-221 )**

Reviewer #2: The work described in this manuscript addresses an important issue of designing a cell-seeded cardiac patch to improve cardiac function. Successful development of such a system could improve cardiac function in adults after ventricular rupture. The limited results in this study show that biodegradable patches implanted at the left ventricular apex with or without cardiomyocytes did not improve cardiac function. The reason for this lack of effect could not be clearly identified due to limitations of the study design.

The authors provide no assessment of cardiomyocyte function or organization prior to implantation, so it unclear whether the implanted cardiomyocytes had any contractile function at the time of implantation.

Cardiac progenitor cells purchased from CDI, thawed, and directly sprayed on bioabsorbable material. This time, it was Cardiac progenitor cells, not a differentiated cardiomyocyte, so it did not have a contractile function.

The small sample size in each group (1 and 2 month time points had 2 animals in each group and the 6 and 9 each had 3 animals in each group) precluded detection of differences between the two groups except at 8 weeks for ejection fraction. The sample size is much smaller than the authors used in their prior study, reference 11.

**Thank you for pointing out. Small sample size is one of the limitations. This study is still at a preliminary stage, and only examines how seeding iPS cells with bioabsorbable material contributes to myocardial regeneration.** **In the future study, we would like to perform power analysis and increase the number of samples. (Page 18 line 298-302)**

The histological analysis of the implants did not examine for the presence of cardiomyocytes or vascularization in the patches. Thus, the reason for the difference in ejection fraction due to cell seeding could not be evaluated.

**Thank you for pointing out. When statistical processing was actually performed using the AVOVA, there was no significant difference in the ejection fraction. We now include in the manuscript quantification of vascularization using IHC against CD31 and found that there was a greater number of new blood vessels in the seeded group. (Figure 6) Cardiomyocytes on the patch were also described in Figure 5C. (Page 13 Line 207-221)**

Specific Comments
1. Figure 4. Horizontal axis label is needed for each panel. **Horizontal axis labels are now included for all panels.**

2. Figure 4. Were the changes in LV systolic and diastolic volumes over time significant? The statistical methods used indicate that t-tests were used, but the results in Figure 4 indicate that a two-factor ANOVA (time and treatment) should be used to evaluate trends with time.

**We performed statistical processing with ANOVA and there was no longer a significant difference between groups at the 2M time point. The manuscript has been updated to reflect this change. (Page 12 line 190-194)**

Reviewer #3: This study tested the feasibility of applying stem-cell patches with a biodegradable substrate to damaged ventricular tissue to enhance long-term remodeling. Engineered tissues for cardiac repair are evolving and in-vivo studies such as are valuable results that may help the field advance toward human use.

Although the goals of the study are modest, and methods used to quantify histological changes are limited, the results do provide useful information and show that this type of tissue engineered patch with human iPS cells and some type of biodegradable substrate can integrate with the native myocardium, albeit with limited functional outcomes at the present time.

A major limitation of the study is that the application of these patches, as stated in the introduction, can help with tissue remodeling in situations such as ventricular aneurysm, but the studies were done in a much different ventricular trauma injury model. The acute injury response to the procedure here is much different than the human diseases mentioned in the introduction and as rationale for the study. This difference is not discussed in the paper.

**Thank you for pointing out. Certainly, the indication described in the introduction is the creation of patches for use after human myocardial infarction. This study is still at a preliminary stage, and only examines how seeding iPS cells with bioabsorbable material contributes to myocardial regeneration. (Page 18 line 298-302) We believe that this model of rat is useful for evaluating remodeling for about one year. Also many paper exist to attach these iPS patch on the above of LV. But there is no report of survival experiments in rats which simulating the left ventricle, that is, simulating VSP or left ventricular rupture, and we consider the importance of this research on the method of surviving the rat for one year. (Page17 line276-279)**

1.The methods should describe how the microscopic imaging were analyzed and what exactly was quantified, including sample sizes.

Also, 2. information on orientation of the sections, since cardiac and scar tissue, the patch and the geometry of the wall are all have direction-dependence structure.

Finally,3. better quantification of the cell numbers and degree of calcification should be done in addition to the qualitative descriptions in the results.

**Thank you for pointing out.**

1. **We have added details on the analysis method of the microscopy. (Page 10 line161-Page11 line172)**

**2. Details have been added to the section. After the sacrifice, the left ventricle was separated from the heart by axial dissection, and a sagittal transection centered on the apical patch was performed. (Page10 line156-157)**

**3. Regarding the area of calcification, there was no statistically significant difference between Seeded (N = 6) 16.0 ± 5.2% vs Unseeded 8.14 ± 3.3% (N = 6) (P = 0.09).(Figure 6C-2) We stained CD31 to check for angiogenesis. There is statistically significant differences at 6M timepoint (N=3). It is defined as above the patch and near the anastomosis. (Page 13 line 207- 221) (Figure 6C-3)**

Part of the study is the examine the biodegradability of the patch substrate, but this was not quantified, and only vaguely described in the paper. How do we know the that “some patch material still remains and is covered by collagen” or that it actually degrades? Some kind measure to back up these statements would be useful.

**Thank you for pointing out. We characterized scaffold degradation and neotissue formation using polarized light images of Picro-Sirius Red (PSR) stained sections from Rat Patch obtained 1 M, 2M, 6M,9M after implantation, which revealed both the degradation of PGA fibers and the deposition and maturation of collagen fibers. The PGA fibers remained highly organized within the scaffold at 1M after implantation, but had thinned and begun to show evidence of early fragmentation at 2M. Only rare thin individual fragments of the PGA fibers were visible by 6M after implantation. (Page 12 line 200-204, Figure 5B)**

The quantitative results are likely under-powered, with small sample sizes (n=2 or 3). Have the authors done any power analysis or other statistical approaches to determine if the numerical results (echo studies) are actually statistically valid? Changes in LV function is one of the stated goals of the study, and it is hard to interpret the functional results with these sample sizes.
**The small sample size is a limitation of this preliminary study. Power size was not verified. (page 18 line 298-302) A new statistical analysis was performed using ANOVA. (page11 line 175-178)**

The conclusions paragraph can be improved. The first and third sentences are not conclusions. The second sentence is the only actual statement of a conclusion, and certainly does not summarize the main findings of the paper.

**Thank you for point out. We have modified the conclusions. (Page19 line313 -317)**

Minor:

Please include page numbers for easier referencing. Also, please use indentation or spacing for new paragraphs.

**Thank you for pointing out. The manuscript now includes page numbers.**

Abstract, last sentence: “differentiate into cardiomyocytes survive”, correct wording.
**Thank you for pointing out.**

**This wording has been changed to “Seeded hiPS-CPCs did not differentiate into cardiomyocytes, but the greater number of new blood vessels in seeded patches suggests the presence of cell seeding early in the remodeling process might provide a prolonged effect on neotissue formation.” (page 4, Line 62-67)**

Intro, line 4: “cell attachment or migration” migration of what cell type?

**This line has been modified to specify macrophage migration. (Page 5 Line 72-74)**

Intro, line 14: “Litations”, Limitations??

**The spelling of this word has been corrected to “limitations”(page 5 Line 83)**

The discussion starts with references to post-infarction remodeling, but the current study does not use that model. It would be better for the rationale for the current experiments to be better related to the same type of remodeling that would occur with the current animal model. Why is “infection” mentioned, when this is not part of the current study (but durability and biocompatibility are)?

**Thank you for your comments and great questions to our experiments. When we try to create infarction model with the additional incision of left ventricular apex for placing the patch in rats, animal survival rates were low following the procedure. Therefore, we simplified the model to utilize left ventricular apex incision to implant our patches in the blood stream. (Page17 line 271-274)**

**In general, cardiac surgeons have concerns about implanting foreign materials in the heart as infective endocarditis can be a lethal complication after open heart surgery. Even now, some surgeons inevitably use Dacron patch or PTFE patch to repair for ventricular septal rupture after myocardial infarction. These clinical demands motivated us to develop new bioabsorbable materials for use in cardiac surgery. This experiment is initial attempt to develop new cell-based materials. (page 14 line227- 237)**

Discussion, first para: “sustain regular rhythm”, why mention this is such a short “intro” in the discussion, when it is not part of the current study?

**Thank you for pointed out. As you mentioned this is not part of the current study so we eliminated this sentence.**

Discussion: “In the past, heart tissue was considered non-renewable and the heart was considered the final organ”. Wording is awkward.

**This section has been modified.**　**(page15 line246)**

Discussion: “….to create a pulsation model.” Not clear what this type of model refers to.
**This section has been modified (page16 line269)**

Discussion: “and host species leading to a non-sychronized heartbeat” not-clear that this is the case or that this would happen here. Also typo in “synchronized”

**This section has been modified.**　**(page18 line298-page19 line310)**
